# Metabolic signatures of *Arabidopsis thaliana* abiotic stress responses elucidate patterns in stress priming, acclimation, and recovery

**DOI:** 10.1007/s44154-022-00034-5

**Published:** 2022-02-15

**Authors:** Yuan Xu, Dana M. Freund, Adrian D. Hegeman, Jerry D. Cohen

**Affiliations:** 1 Department of Horticultural Science and the Microbial and Plant Genomics Institute, University of Minnesota, MN Saint Paul, USA; 2 Department of Plant and Microbial Biology, University of Minnesota, MN Saint Paul, USA

**Keywords:** Abiotic stress, *Arabidopsis thaliana*, LC-HRMS, Metabolomics, Plant stress acclimation, Priming, Recovery

## Abstract

Temperature, water, and light are three abiotic stress factors that have major influences on plant growth, development, and reproduction. Plants can be primed by a prior mild stress to enhance their resistance to future stress. We used an untargeted metabolomics approach to examine *Arabidopsis thaliana* 11-day-old seedling’s abiotic stress responses including heat (with and without priming), cold (with and without priming), water-deficit and high-light before and after a 2-day-recovery period. Analysis of the physiological phenotypes showed that seedlings with stress treatment resulted in a reduction in fresh weight, hypocotyl and root length but remained viable. Several stress responsive metabolites were identified, confirmed with reference standards, quantified, and clustered. We identified shared and specific stress signatures for cold, heat, water-deficit, and high-light treatments. Central metabolism including amino acid metabolism, sugar metabolism, glycolysis, TCA cycle, GABA shunt, glutathione metabolism, purine metabolism, and urea cycle were found to undergo changes that are fundamentally different, although some shared commonalities in response to different treatments. Large increases in cysteine abundance and decreases in reduced glutathione were observed following multiple stress treatments highlighting the importance of oxidative stress as a general phenomenon in abiotic stress. Large fold increases in low-turnover amino acids and maltose demonstrate the critical role of protein and starch autolysis in early abiotic stress responses.

**Supplementary Information:**

The online version contains supplementary material available at 10.1007/s44154-022-00034-5.

## Introduction

Stress in plants has been described as any condition that causes a change in optimal growth, development, and reproduction (Levitt, 1972; De Oliveira, 2019). Stress can be classified as abiotic and biotic (Pandey et al., 2017) with abiotic stress caused by nonliving factors in the environment such as temperature, the supply of water, and light intensity (Levitt, 1972; Boscaiu and Fita, 2020). Abiotic stress is especially constraining to plants because, unlike animals, plants are sessile and are thus particularly dependent on environmental factors (Rizhsky, 2004). Even though abiotic stresses increase damage risk, prior priming treatments can transiently enhance or extend plant overall stress tolerance against future stress (Levitt, 1972; Hallberg et al., 1985; Thomashow, 1999; Kanjariya and Parihar, 2017; Mauch-Mani et al., 2017). For example, plants can tolerate normally lethal high or freezing temperatures when they are pre-exposed to nonlethal high or low temperatures respectively (Guy et al., 1985; Guy, 1999; Hallberg et al., 1985; Thomashow, 1999). Priming consists of three stages: the priming phase, the post-challenge primed state, and the transgenerational primed state (Conrath, 2011). This priming effect has been studied regarding epigenetics (Crisp et al., 2016), transcriptomics (Avramova, 2015; Firtzlaff et al., 2016), and proteomics (Li and Liu, 2016). Recent metabolomics studies on the priming effect revealed metabolic changes in amino acids, hormone conjugates, and sugars during priming (Balmer et al., 2015; Wang et al., 2018; Tugizimana et al., 2019; Shehu et al., 2019). However, the comprehensive metabolome changes of the priming effect are still far from being fully elucidated.

Several previous metabolomics studies analyzing plant abiotic stress responses have been reported in recent reviews (Carrera et al., 2021; Ghatak et al., 2018; Nephali et al., 2020) including heat, cold, freezing, water-deficit, and high light exposure. Still, little has been done to integrate these stress responses to create a uniform understanding of shared and specific metabolic stress responses that are combined to mount a unified response to multiple stressors as they often are encountered in nature. In this present work we aimed to combine conditions and duration of stress treatments from numerous recent studies (summarized in Supplemental Table S1) to form a best compromised set of treatments that allowed reasonable comparisons between different types of abiotic stressors.

The goals of this study were to contrast the metabolic changes induced by heat, cold, water-deficit, and high light in *Arabidopsis thaliana* 11-day-old seedlings with and without priming and a 2-day-recovery treatment to find primary metabolic changes that are shared and specific stress metabolic signatures for different stressors. Similarly, the metabolic consequences of priming and a 2-day-recovery period were also examined and compared to define changes in common and the differences in the metabolic responses to stress priming and recovery preceding and following abiotic exposure. Together, these studies have provided insight into metabolic responses that allow plants to tolerate and recover from a number of different environmental changes.

## Results

### Calibration of the physiological impacts of stress treatments

Prior to metabolomic analysis, stress treatment conditions (Table 1) were carefully selected from other studies to provide a significant sub-lethal impact on Arabidopsis seedling physiology. Over-all fresh weight, hypocotyl, and root length before and after a 2-day-recovery period were monitored to provide metrics for the physiological impact of each stress treatment. Seedlings showed significant decrease (*P* < 0.05) in fresh weight compared with control under HP (28% decrease), BH (46% decrease), WD (69% decrease), BHR (43% decrease), WDR (49% decrease), and in all cases the average values were similar or less than their respective controls, as one would anticipate from exposure to sub-lethal stress conditions (Supplemental Fig. S1A). Seedlings also showed significant decrease in both hypocotyl length, under WD (25% decrease), CPR (31% decrease), and HPR (28% decrease), BHR (23% decrease), and WDR (36% decrease) (Supplemental Fig. S1B), and root length, under CP (16% decrease), HP (27% decrease), BH (21% decrease), WD (38% decrease), HL (14% decrease), HPR (27% decrease), BHR (16% decrease), and WDR (17% decrease) (Supplemental Fig. S1C).

**Table 1:** Abiotic stress conditions

Stress treatment	Conditions	Time	Reference^**a**^
Basal cold (BC)	4 °C for 3 h	9:00 AM	Kaplan et al., 2004
Cold pretreatment (CP)	4 °C for 3 h, 22 °C for 1 h, then −15 °C for 1 h	9:00 AM	Kaplan et al., 2004
Basal heat (BH)	45 °C for 5 h	9:00 AM	Kaplan et al., 2004;
Heat pretreatment (HP)	38 °C for 1.5 h, 22 °C for 2 h, then 45 °C for 5 h	9:00 AM	Kaplan et al., 2004; Larkindale, 2005
Water deficit (WD)	remove seedling grown on nylon membrane from medium and desiccate in air for 2 h	9:00 AM	Li et al., 2008
High light (HL)	902 μmol m^−2^ s^−1^ metal halide light for 1 h	9:00 AM	Rossel et al., 2002
**Recovery treatment**	**Recovery Conditions** ^**b**^		
Basal heat (BHR)	plates of seedlings moved from stress to control chamber for 48 h	2:00 PM	Olas et al., 2021
Heat pretreatment (HPR)	plates of seedlings moved from stress to control chamber for 48 h	5:30 PM	Olas et al., 2021
Basal cold (BCR)	plates of seedlings moved from stress to control chamber for 48 h	11:00 AM	Zhang et al., 2004
Cold pretreatment (CPR)	plates of seedlings moved from stress to control chamber for 48 h	2:00 PM	Guo et al., 2021
Water deficit (WDR)	seedlings transferred back to agar medium in control chamber for 48 h	11:00 AM	Hernandez et al., 2002
High light (HLR)	plates of seedlings moved from stress to control chamber for 48 h	10:00 AM	Richly et al., 2003

^a^Source from which stress conditions, with minor modifications, were derived

^b^All recovery treatments were identical to the above stress treatments, but were sampled after a 48 h recovery period at ‘control conditions’ (22 °C, 16 h-light/8 h-dark photoperiod with lights on at 8:00 AM, and ~ 80 μmol m^−2^ s^− 1^ from cool-white fluorescent lights)

### Untargeted metabolomics of abiotic stresses and recovery

**Fig. 1:**
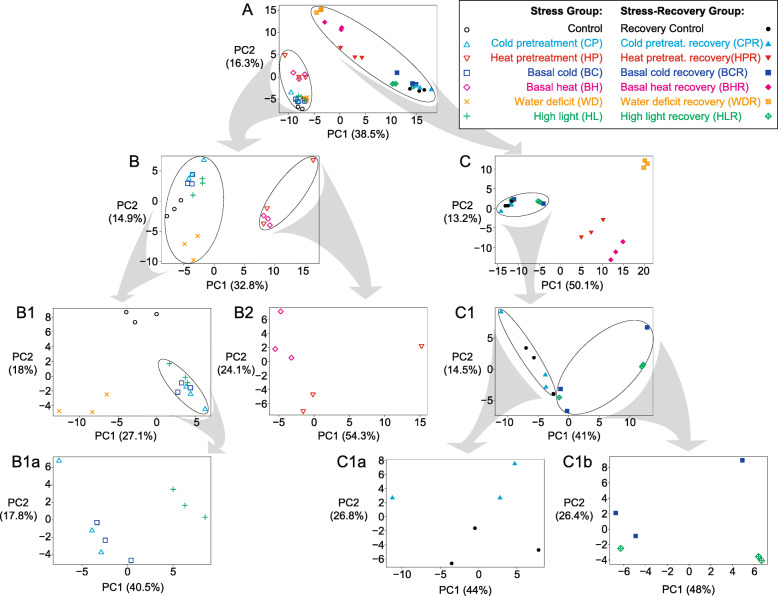
Unsupervised clustering of treatment groups with iterative reanalysis of subgroups. Scores plots are shown from the PCA analysis of untargeted LC-MS data derived from different seedling stress and recovery treatments. PCA analysis was reperformed using subgroups identified from prior treatment clusters. Symbols and colors correspond to treatments in stress and recovery groups given in the key. (**A**) All groups. (**B**) Stress groups. (**C**) Recovery groups. (**B1**) Control, CP, BC, WD, HL subgroup. (**B2**) BH and HP subgroup. (**C1**) Control recovery, CPR, BCR, and HLR subgroup. (**B1a**) CP, BC, and HL subgroup. (**C1a**) Control recovery and CPR subgroup. (**C1b**) BCR and HLR subgroup

A total of 1180 metabolic features and their intensities were measured from HILIC LC-HRMS analyses of extracts from 11-day old seedlings subjected to the stress, recovery, and control conditions as detailed in Table 1. Each metabolic feature consists of 1) an accurate *m/z* (error ~ 2 ppm); 2) charge (positive or negative); and 3) a chromatographic retention time. Arabidopsis seedlings were subjected to each experimental condition and analyzed in triplicate to generate an ion intensity value matrix consisting of ion abundance measurements for each feature and each replicate of each treatment. The data matrix was subjected to PCA in a hierarchical manner to assess the extent of divergence within metabolite populations between treatments (Fig. 1). First, PCA of datasets from all conditions was performed (Fig. 1A), which resulted in two large clusters consisting of the stress and stress-control group well-separated from the recovery and recovery-control group. This clustering pattern indicated that significant metabolic changes occur during the 2-day growth recovery period regardless of treatment. Re-analyses of stress and stress-control (Fig. 1B) and recovery and recovery-control (Fig. 1C) subgroups were performed to separate these groups into additional sub-clusters.

In the stress subgroup, both HP and BH heat stresses are clustered together and are well separated from the other stresses and control, indicating that heat stress has the most dramatically different metabolic response within this group (Fig. 1B). Additional reanalysis of subgroups (HL, WD, CP, BC, and control) in Fig. 1B1 shows the control is well separated from the stress groups in PC2 (18%). WD is also well separated from the other stresses in this subgroup, while CP, BC and HL have more similar metabolic patterns. PCA of BH and HP subgroup (Fig. 1B2) showed these two sets of samples are not well separated in either PC1 or PC2, indicating priming treatment of heat stress has a relatively minor effect right after the stress treatment. Subsequent PCA of the CP, BC, and HL subgroups (Fig. 1B1a) showed that BC and CP cluster together but are separated from HL, indicating that high light stress can be metabolically distinguished from cold stress, and that priming treatment for cold, like that observed for heat, has little significant difference from the unprimed treatment right after the stress treatment.

In the PCA of the recovery subgroup, WDR, BHR, and HPR were well separated from another subgroup composed of CPR, BCR, HLR, together with the recovery control. These results indicate that water-deficit and heat have the most distinct metabolic responses (Fig. 1C), and that the priming treatment for heat has a large and significant impact during the 2-day-recovery. Subgroup PCA (Fig. 1C1) showed CPR and the recovery control are separated from BCR and HLR. Additional subgroup PCAs (Fig. 1C1a and Fig. 1C1b) showed minor separation of a CPR and the recovery control subgroup, and a BCR and HLR subgroup. These results show how the priming effect is apparent after the 2-day-recovery even when it is not obvious immediately following the initial stress treatment. Additionally, CP treatment is similar to the control in contrast to BCR. BC is, interestingly, more closely related to HLR. While it is known that Arabidopsis is more resilient to CP (Leuendorf et al. 2020), given the PCA results, it appears that priming leads to a faster metabolic recovery from cold stress without causing a dramatically different metabolic response to initial cold stress conditions.

### Targeted metabolite analysis and hierarchical clustering

From the untargeted data matrix, feature abundance values for 3285 conditions that had at least a 2-fold change from control (significance assessed by ANOVA with *P* < 0.05) were identified for each treatment. Metabolites that were identified from these features based on elemental composition assignment of accurate mass values in one or more stress conditions were selected for further targeted analysis to provide a better representation of the central metabolic stress response. Ultimately the putative identities of 47 metabolites were confirmed by: 1) comparison of chromatographic retention by co-injection; and 2) by comparison of MS/MS fragmentation behavior (Supplemental Figs. S2.1 and S2.2) with standard compounds (Supplemental Table S4). These confidently identified metabolites included: 21 amino acids (including 18 of the proteinogenic amino acids); 7 sugars, 7 TCA cycle intermediates, 4 glycolytic pathway intermediates, 4 purine metabolites, oxidized and reduced glutathione, urea, and shikimic acid. After metabolite identities were confirmed, their corresponding LC-HRMS peaks were manually integrated in both positive and negative ionization modes and the fold change in the average intensities across replicates between stress and control samples was calculated. Fold-changes in intensities for metabolites observed in both ionization modes were averaged if they were deemed indistinguishable by ANOVA, otherwise the values derived from the most intense signals were used on the assumption that the values derived from lower signals were lower due to ion-suppression. These confirmed metabolite abundance fold-change values (Supplemental Table S3) and treatments were then clustered by hierarchical clustering analysis (HCA) to provide relationships between stressors, priming, and recovery using this targeted central metabolic dataset. HCA analysis was performed using the complete linkage with the maximum distance measurement method based on De Souza et al. (2006) and Edelbrock (1979). We also tested complete linkage using other distance methods, including Euclidean, Manhattan, Minkowski, Ward, and McQuitty, with comparable results (data not shown). Features from 2833 treatment conditions that had at least a 2-fold change from control (significance assessed by ANOVA with *P* < 0.05) and that we did not positively identify are reported in Supplemental Table S5.

**Fig. 2:**
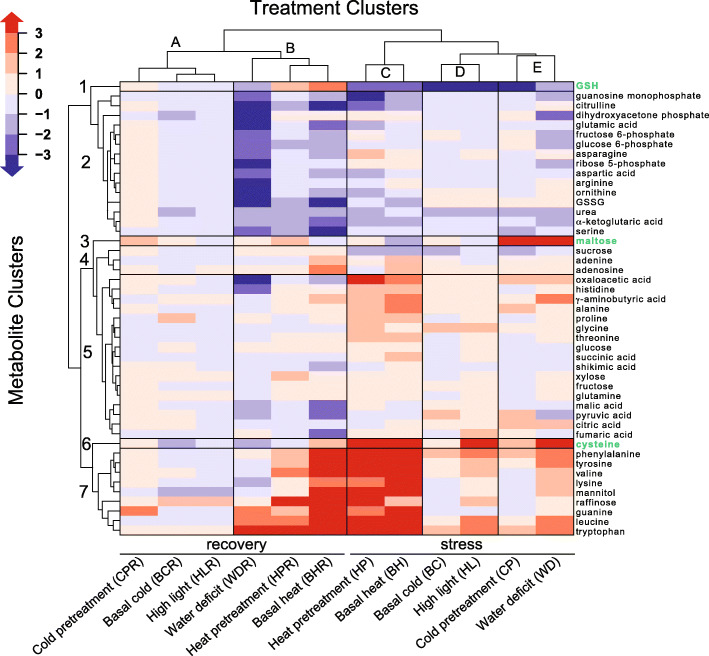
Heat map generated from a two-way hierarchical clustering analysis (HCA) of changes in metabolite abundance vs. treatment. The scale bar (top left) reflects the log_2_-transformed of the average fold changes derived from both positive and negative mode observations as reported in Supplemental Table S3. Clusters were generated using the complete linkage and maximum distance measurement. Five treatment clusters were labeled **A**-**E**, and seven metabolite clusters were labeled 1–7. Metabolites by themselves in clusters 1, 3 and 6 are highlighted in green

A heatmap of the HCA indicates five distinct clusters for the six stress and six recovery treatments (Fig. 2) identified as A-E, as well as seven clusters of metabolites numbered 1–7. Treatment cluster A included CPR, BCR, and HLR, which displayed only modest changes in all metabolite clusters, while cluster B, composed of treatments WDR, HPR, and BHR displayed decreased abundance in cluster 2 and increased abundance in cluster 7. Cluster C consisted of treatments HP and BH and showed a decreased abundance in metabolites within cluster 1 and a dramatic increase in abundance of metabolites from clusters 6 and 7 and a modest increase from cluster 5. Cluster D included treatments BC and HL and it displayed a dramatic decrease in abundance of metabolites in cluster 1, and a modest increase in metabolite abundance from clusters 6 and 7. Cluster E contained treatments CP and WD and displayed increased abundance of metabolites in clusters 3 and 6 and decreased abundance in cluster 1.

## Discussion

### Experimental design considerations

We conducted a simultaneous analysis of the effects of abiotic stress conditions, including cold, heat, water-deficit, and high light on the *Arabidopsis thaliana* metabolome, and monitored primary metabolite variations accompanying priming, for cold and heat stress, and recovery for each stress treatment. Stress treatment conditions (Table 1) were selected from prior studies to provide a significant but sub-lethal stress on Arabidopsis seedlings. A period of 48 h was selected for recovery based on previous studies of heat stress and heat stress priming recovery in Arabidopsis (Olas et al., 2021), drought and cold stress recovery in maize (Guo et al., 2021), freezing stress recovery in *Brassica napus* (Zhang et al., 2004), salinity stress stress recovery in pea (Hernández et al., 2002), and high light stress recovery in Arabidopsis (Richly et al., 2003). In each type of stress response 48 h provides a compromise in phenotypic stress recovery without complete readaptation to unstressed growth conditions, and it allows plant materials to be harvested at the same time in their photoperiod so that diel variation can be minimized.

While much previous work has shown changes in the accumulation patterns of specialized or secondary metabolism in response to stress conditions (Carrera et al., 2021), in this study we intentionally chose to focus on the perturbations of primary metabolism caused by each abiotic stress in parallel. Our specific interest in primary metabolism is because these metabolic reactions underlying plant growth are ongoing prior to the onset of stress, they are then physically perturbed by abiotic stress conditions, but then must be reestablished for continued growth. We are interested in how regulation and maintenance of homeostasis in primary metabolism is maintained or adjusted through the phases of stress and recovery.

In order to be able to measure common pools of primary metabolites and avoid some of the chemically harsh conditions used for GC-MS derivatization strategies we used LC-HRMS and HILIC rather than reversed phase chromatographic media (Zhang and Watson, 2015). We identified 47 primary metabolites, confirmed their identities using authentic standard compounds, and mapped their abundance changes relative to un-stressed controls onto a simple metabolic model using a graphical method that allows direct comparison of abundance changes across all the stress and stress-recovery treatments for each metabolite.

### The full extent of stress-induced metabolic perturbations only becomes apparent after recovery

By examining the untargeted analysis data and hierarchical PCA of the stressed and recovering seedlings we can get a general observation of the extent of metabolic perturbation for each condition from its control and see which conditions cluster together and thus are metabolically similar. In Fig. 1, within the stress group, we see that HP and BH are most distinct from control followed by WD. BC and CP cluster together and are similar but distinct from HL. The control cluster in the stress group is closest to BC, CP, and HL, yet clearly distinct (Fig. 1B1). In the stress-recovery group, the separation between the recovery control, CPR, BCR, and HLR are less clear, and the CPR appears to be clustered closely with the control as compared with the basal cold recovery and BCR and HLR. For heat stress recovery, both HPR and BHR are well separated from the control recovery cluster. WDR also appears to be metabolically distinct.

It is interesting to see how BC, CP and HL cluster together in this analysis. It is well known that low temperatures decrease the light intensity threshold at which plants experience photodamage (Szymańska et al., 2017). Maxwell et al. (1995) investigated the relationship between cold and light in the green alga *Chlorella*. They found *Chlorella* acclimated to cold have higher xanthophyll content, higher ratio of chlorophyll a/b, and lower chlorophyll content. The same group also found photosynthetic acclimation to cold could be used to predict freezing tolerance to winter annuals and algae (Huner et al., 1998). Common elements involved in the sensing and transduction of light and temperature signals have also been identified *in planta* (Legris et al., 2016, 2017).

Some of the most interesting information from the untargeted analysis comes from the differences in metabolic stress responses before and after recovery. Note the lack of separation between the basal and pretreated plants under both heat and cold conditions in the stress group that become well resolved in the stress recovery group. This is most dramatic with the heat stresses but also apparent in the cold stresses. It appears that the pretreatments in both cold and heat have little metabolic impact in the time frame of the initial stress experiments (3.5 h for heat, and 4 h for cold pretreatments, see Table 1) yet trigger significant metabolic changes during recovery. Given previous descriptions of cold and heat stress response kinetics and the sampling times (3 and 5 h respectively) for the BC and CP plants, it is likely that gene transcription promoted by cold stress has not started to have a significant impact on metabolite abundance (see Fig. 1B1a). While for BH and HP, which have both later sampling times (5 and 8.5 h respectively) and faster stress response kinetics, show more distinct clusters in the PCA scores plots (see Fig. 1B2) (Kaplan et al., 2004). Pretreatments are often thought to prime a stress response so that metabolic changes can occur that will help protect the plant from more extreme forms of the same stress. Indeed, this does appear to speed the metabolic recovery processes as CPR metabolism is nearly indistinguishable from that of the recovery control, and the HPR cluster lies between the BHR and the recovery control. So while pretreatment seems to show little impact on metabolism within the initial stress trial it does appear to allow metabolism to recover more quickly than with the basal stress condition alone, perhaps explaining what was previously observed with physiological stress recovery (Li et al. 2021).

Another big difference between the stress and recovery groups is observed between WD and WDR. In the stress group, WD clusters loosely (Fig. 1B) with the control, BC, CP & HL but can be distinguished with subsequent rounds of PCA (Fig. 1B1). In the recovery group, WDR results in the greatest metabolic differentiation, even surpassing BHR and HPR. This result is consistent with plastid proteomic analysis (Tamburino et al. 2017) that also revealed profound alterations under recovery from a drought stress period.

### Targeted metabolites highlight stress signatures in central metabolism

The targeted dataset is obviously a small subset of the compounds studied in untargeted analysis, thus the general observations from the larger untargeted dataset are focused to some extent, although not dissimilar when compared within the treatment cluster tree in Fig. 2. Perhaps more importantly, from this analysis we can start to understand the biochemical underpinnings of the stress and recovery responses.

For example, we found that many metabolites change in abundance in similar patterns in response to the different stress and recovery conditions. Hierarchical clustering analysis (Fig. 2**)**, shows metabolites in clusters 2, 4, 5, and 7 exhibited similar response patterns across different abiotic stresses within each cluster. Cluster 2 contains 15 metabolites including 7 amino acids (Cit, Glu, Asp, Asn, Arg, Orn and Ser), 4 sugar phosphates, a nucleotide (GMP), oxidized GSSG, and urea. These results are consistent with recent studies showing a close relationship between amino acid metabolism and stress responses (Batista-Silva et al. 2019). The compounds in this cluster show moderate changes (less than 2-fold up or down) to most conditions including CPR, BCR, HLR, BC, HL and CP, but show dramatic decreases under WDR and BHR, with mixed responses to HP and WD. Cluster 7 consists of nine metabolites, including six aromatic amino acids (Phe, Tyr, Trp, Val, Leu and Lys), a sugar alcohol (mannitol), a trisaccharide (raffinose), and guanine. Cluster 7 increased in HPR, BHR, HP, BH, HL and WD, generally decreased in BCR and HLR with more mixed responses in CPR, WDR, CP, and BC. Clusters 4 and 5 are more variable with fewer remarkable patterns that fall between the extremes of groups 2 and 7. Cluster 5, for example, shows a moderate increase in abundance under HP and BH and moderate decrease under WDR with little change in CPR, BCR and HLR and mixed responses under HPR, BHR, BC, HL, CP, and WD. Three metabolites, reduced GSH, Cys, and maltose, showed unique and dramatic changes in abundance across the panel of stress conditions that is discussed in more detail below.

### Similar patterns with prior metabolomic abiotic stress studies

There are multiple challenges in making generalized comparisons between any two metabolic stress response studies including differences in the stress response timing and stress severity as well as differences in responses between plant species or even in different developmental stages, tissue types, or growth regimes for the same species. Changes in metabolite abundance across stresses show broad similarities with numerous prior studies of individual stress responses. For heat stress, increases in amino acid pools (including Ala, Asn, Gly, Leu, Lys, Thr, Tyr, and Val) and other metabolites such as γ-aminobutyric acid, raffinose, xylose, shikimic acid, and succinic acid, and a decrease in Asp and glucose 6-phosphate have been documented in 3-week-old Arabidopsis exposed to continuous 40 °C temperatures over several days (Kaplan et al., 2004). For cold stress, with either 3-week-old or 6-week-old Arabidopsis grown at 4 °C continuously, two studies showed similar results to ours including increases in maltose and amino acids (Phe, Gly, Ala, Leu, Val and Orn), though both studies also show variation in the timing of metabolite abundance changes (Kaplan et al., 2004; Caldana et al., 2011). For water-deficit stress, one study used aerial portions of 4-week old Arabidopsis plants, which were removed and allowed to dessicate on paper; this resulted in increases (similar to our study) in the abundance of maltose, amino acids (Ala, Gly, His, Leu, Val, Tyr, Trp, Phe, Lys, Thr, Pro), γ-aminobutyric acid, raffinose, xylose, and the decrease of Asp and pyruvate (Urano et al., 2009). Lastly, for two studies of high light stress with either 3-week or 6-week-old Arabidopsis grown with light intensities of either 700–1000 or 400 μmoles m^− 2^ s^− 1^ PAR showed similarities with our study including an increase in abundance of amino acids (Phe, Gly, Pro, Gln), raffinose, glucose, and fructose (Wulff-Zottele et al., 2010; Caldana et al., 2011).

### Apparent differences with previous metabolomic stress studies

Significant discrepancies are also apparent between our study and previous metabolic profiling studies of abiotic stress responses. Some of these differences are likely due to differences in analytical methodology employed in each study. For example, metabolite extraction followed by methoxyamination and trimethylsilylation derivatization, and GC-MS analysis is commonly used in plant metabolomics studies, but it will not provide usable quantitative information for some chemically unstable or high boiling point analytes so that cysteine, tryptophan, α-ketoacids, or peptides such as glutathione will be absent. The method employed in this study is rapid and chemically gentle (plants are frozen in liquid nitrogen with an inert atmosphere, there are no pH extremes, low temperature was maintained, and inert extraction solvent was used) so that chemically and oxidatively labile analytes are quantifiable. As a result, very few previous metabolomics studies document the large changes that we observed in Cys, GSH, GSSG, Trp, oxaloacetate or several of the phosphorylated glycolytic intermediates. Wulff-Zottele et al. (2010) did report Cys and glutathione (GSH + GSSG) as part of their study, but these were obtained using an HPLC method and were reductively treated prior to analysis. Other studies using targeted methods focus on the roles of Cys, GSSG and GSH specifically in plant stresses and will be discussed below.

Another source of inconsistencies with other studies relates to differences in sampling time following application of stress conditions. Abiotic stress responses are often thought of as occurring in phases where initial metabolic changes occur first followed by transcriptionally driven adaptations that follow several hours after stressful conditions commence. Kaplan et al. (2007) observed rapid changes in metabolite abundance in the first 12 h of Arabidopsis subjected to cold stress (at 4 °C). This is followed by a second phase from 12 to 96 h where metabolism is directed through newly transcribed genes in response to cold perception. Our study examined BC stress after growth at 4 °C for 3 h, which is well before the transcriptional phase of the response. Kaplan et al. (2004) observed that the elevated temperature stress (40 °C) response occurs much more rapidly than the cold stress response, with the initial rapid metabolic phase taking under 1 h and the transcriptionally driven adaptation taking 4–6 h. Given that our stress treatments fall between 1 and 8.5 h for all our stress conditions, the differences in stress response speed between each stress will result in sampling in different phases of each response. An 800% increase in maltose abundance in Arabidopsis grown at 40 °C was observed after 30 min by Kaplan et al. in their 2004 manuscript, but after 4 h the abundance dropped down to 25% of the control. Because we wanted to minimize variation and perform our stress experiments in parallel on the same day, the addition of a longitudinal experimental design element that would have allowed us to capture the kinetics of each stress response was impractical. As a result, some transient metabolite abundance changes may have been missed in the more rapid stress responses, like BH and HP, while later phase transcriptionally driven changes may not yet have begun to take place in the slower stress responses, like BC and CP.

### Metabolic regulation of osmotic stress and ROS

**Fig. 3:**
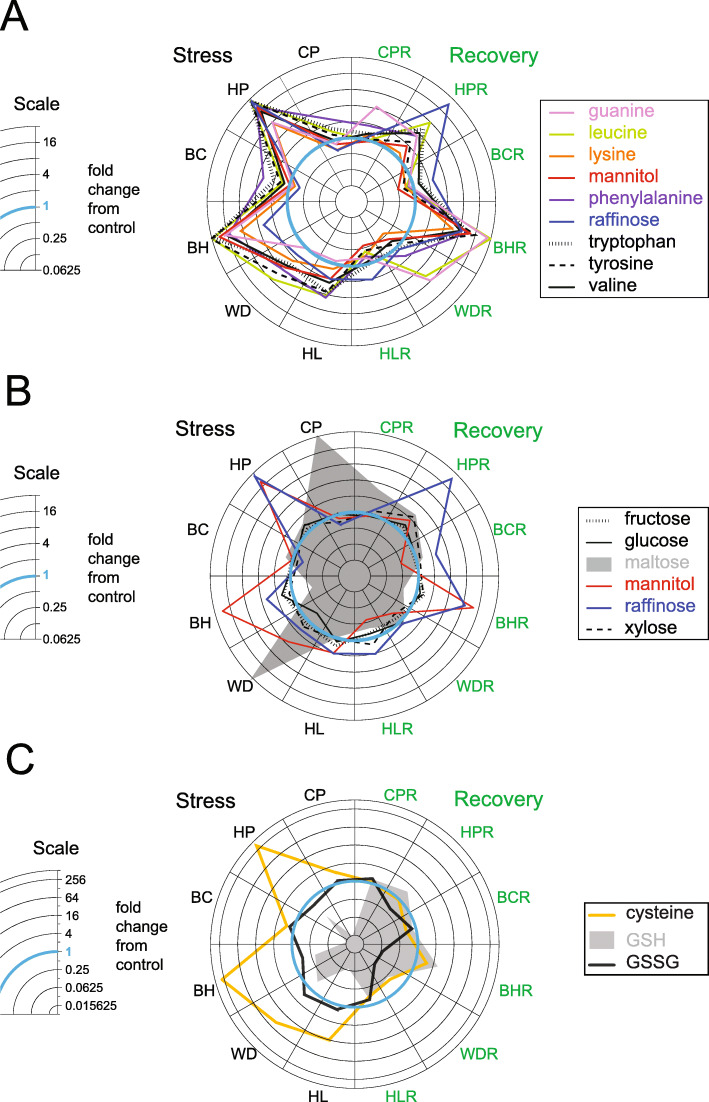
Radial plots comparing the Fold abundance changes for three groups of metabolites observed across all treatments relative to control. Tryptophan, phenylalanine, tyrosine, leucine, valine, lysine, mannitol, raffinose, and guanine are shown in panel **A**; maltose, glucose, fructose, xylose, mannitol, and raffinose are shown in panel **B**; and cysteine, reduced glutathione (GSH), and oxidized glutathione (GSSG) are shown in panel **C**. A ratio change of ‘1’ means that the metabolite abundance was identical for both the treatment and the control and is indicated by the blue circle. Treatments are BC–basal cold stress; BCR–basal cold recovery; BH–basal heat stress; BHR–basal heat recovery; CP–cold pretreatment stress; CPR–cold pretreatment recovery; HL–high light stress; HLR–high light recovery; HP–heat pretreatment stress; HPR–heat pretreatment recovery; WD–water-deficit stress; and WDR–water-deficit recovery

What might be the major driving factors behind these metabolic stress signature similarities and differences? The largest increases in metabolite abundance occur in cluster 7, maltose (cluster 3) and Cys (cluster 6), which together suggest that osmotic and ROS regulation are likely determinants. Figure 3A shows all the cluster 7 metabolites (guanine, Leu, Lys, mannitol, Phe, raffinose, Trp, Tyr and Val) plotted on a radial plot with the different stress conditions arrayed with bilateral symmetry (stress group on the left, recovery group on the right). These metabolites show dramatic increases in abundance across multiple stress and recovery conditions, and all can function as compatible solutes under osmotic stress. Osmotic stress is known to occur in almost all environmental stress conditions including growth under water deficit, high salinity, cold/freezing, or high temperature conditions (Kumar et al., 2021). Seki et al. (2002) found strong relationships between cold, water-deficit, and high-salinity stresses by monitoring the expression profiles of 7000 Arabidopsis genes. Both cold and water-deficit have been shown to activate the expression of genes containing the drought-responsive and abscisic-acid-responsive promoter elements (DRE and ABRE) cis-acting element and drought-responsive-element-binding (DRE-binding) transcription factors DREB/CBF (Liu et al., 1998). Rajashekar and Panda, 2014 reported that water-deficit stress can trigger cold tolerance in Arabidopsis, wheat, oats, rye, and strawberry. Liu et al. (1998) found that water-deficit stress induces the DREB2A gene encoding a DRE/CRT-binding protein, and Sakuma et al. (2006) reported that plants overexpressing DREB2A induced heat shock stress related gene expression and significantly increased thermotolerance. Thus, DREB2A functions in both water and heat shock stress responses. Rizhsky (2004) found that the response to heat and water-deficit combined stress has many unique altered transcripts and metabolic features.

Six of the nine metabolites in cluster 7 are amino acids. It has been proposed that increases in certain amino acids under abiotic stress conditions due to higher protein turnover (Hildebrandt, 2018) possibly resulting from increased protein denaturation and/or damage (Tivendale et al., 2020). While some proteinogenic amino acids (Glu, Asp and Ser) show decreased abundance under stress conditions, it should be noted that Leu, Trp, Lys, Val and Tyr all have some of the slowest turnover rates for amino acids (with half-lives between 100 and 150 h as measured in Arabidopsis leaf tissue; Chen et al., 2011) and thus will not quickly decrease in abundance if amino acids are generated at high rates by proteolysis. Phe has a half-life of ~ 35 h (Chen et al., 2011) but probably has significant flux into phenylpropanoids, while Glu, Asp, Asn and Ser in cluster 2 have some of the faster turnover rates and thus can decrease quite rapidly. Previous studies have shown that osmotic stress can induce the accumulation of branched-chain amino acids through abscisic acid-regulated protein degradation (Huang and Jander, 2017)*.* It thus seems likely that the abundance increase of the cluster 7 amino acids during many of the stress conditions is due to protein autolysis.

Another consideration is the generation of antioxidants to detoxify ROS and prevent oxidative damage during oxidative stress (Kumar et al., 2020). ROS has been suggested to have a dual function in abiotic stress responses such that at high levels ROS are toxic to cells while the same ROS can function as a signal that activates local and systemic plant defenses (Nadarajah, 2020). Aromatic amino acids (Trp, Phe and Tyr) shown in Fig. 3a are precursors for pigments, alkaloids, phytohormones, polyphenolics, and cell wall components and are often effective antioxidants (Dixon, 2001). Figure 3B shows response patterns for glucose, xylose and fructose in cluster 5, mannitol and raffinose in cluster 7 and maltose. Carbohydrates like raffinose (Nishizawa et al., 2008) and sugar alcohols such as mannitol (Chiang et al., 2005) have been reported to improve plant abiotic stress tolerance by acting as ROS antioxidants. Glucose, fructose, xylose, raffinose, and mannitol are also known osmoprotectant molecules during cold, water-deficit, salt, and osmotic stresses (Guy et al., 1992; Fan et al., 1993; Uemura et al., 2003; Rizhsky, 2004; Chiang et al., 2005; Nishizawa et al., 2008). It is notable how little perturbation there is in the abundance of fructose, glucose, sucrose and xylose across treatments, and this may reflect the importance of these sugars in maintaining metabolic homeostasis.

### Changes in Cys, GSH, and GSSG levels in relation to abiotic stress

Figure 3C shows the response pattern for Cys, GSSG, and GSH. GSH is a main low molecular mass plant non-protein thiol compound that is important for regulation of the redox state of the cells (Alscher, 1989). It functions both as an antioxidant and a key regulatory signal in plant defense systems (Szalai et al., 2009). The biosynthesis of GSH consists of two enzymatic reactions (Strohm et al., 1995). First, Glu and Cys form γ-glutamylcysteine. Second, γ-glutamylcysteine and Gly form GSH. Under stress conditions, GSH functions as an antioxidant for ROS via the ascorbate-GSH cycle to form GSSG, resulting in the increase for GSSG and GSSG/GSH ratio (Foyer et al., 1997). Along with its function as an antioxidant, GSH also performs other roles such as thiol disulfide exchange, detoxification, and cell signaling (Hasanuzzaman et al.*,* 2017). Thus, alteration of GSH concentration may function together with the changes in amino acids such as Glu and Gln, which are GSH-dependent through the γ-glutamyl cycle. GSTs can also function in processes activating genes for acclimation, stress tolerance, and pathogen defense response (Wingate et al., 1988).

GSH has previously been reported to play important roles for plant abiotic stress defense responses including chilling and cold (Foster and Hess, 1980), heat (Nieto-Sotelo et al., 1999), water-deficit (Gamble and Burke, 1984), salt (Roxas et al., 1997), high light (Cakmak and Marschner, 1992), and UV irradiation (Strid et al., 1994). Figure 3C shows the radial plots for ratio changes of Cys, reduced GSH, and oxidized GSSG**.** Cys, as one of the precursors of GSH, increased in all the stress groups, especially with a dramatic increase in the HP and BH, with fold changes of 339 and 297 respectively. GSH decreased in all the stress groups, especially in CP, BC, and HL, with ratio changes of 66, 66 and 20, respectively. In contrast, GSSG had minor changes compared to GSH and Cys. The increase of Cys in stress groups may be related to changes in GSH biosynthesis. The decreased GSH in all the stress groups may result from the oxidation of GSH through its function as an antioxidant and this agrees with the reported induction of a steep oxidation of the glutathione pool with high light (Hipsch et al., 2021). However, although the transcripts for both γ-glutamylcysteine synthetase and GSH synthase are often upregulated during abiotic stress (Dorion et al. 2021) it is nevertheless also possible that the biosynthetic pathway from Cys to GSH is blocked during HP and BH, which leads to the accumulation of Cys and decrease of GSH. Cys, GSH, and GSSG all showed minor changes during recovery, indicating resetting is the predominant strategy for glutathione metabolism during recovery.

### Maltose from starch provides an alternative osmotic regulatory strategy

Maltose is the direct product from starch breakdown by β-amylase. The radial plots for ratio changes of maltose and other sugars including glucose, fructose, xylose, mannitol, and raffinose are shown in Fig. 3B. Maltose is elevated in CP and WD up to 33- and 36-fold, but decreased in BH, HL, BHR, and BHL. The increase seen with maltose is likely due to starch degradation since previous work showed that the activity and transcription of β-amylase can be induced during temperature stress (Dreier et al., 1995). Kaplan and Guy (2005) reported maltose accumulated in Arabidopsis by starch degradation following cold shock possibly to protect the photosynthetic electron transport chain and proteins in chloroplast stroma. Rizhsky (2004) reported that both maltose abundance and the expression of starch degradation transcripts were elevated in response to water-deficit and heat combination stress. Maltose was also reported to function as a compatible solute and thus contributed to the protection of plants from freezing stress (Kaplan et al., 2004). It has been suggested that moderate levels of water-deficit stress induce elevated Pro and glycine-betaine, whereas severe levels of water-deficit stress induce sugar accumulation (Hoekstra et al., 2001). In our study, maltose increased in CP and WD by 33- and 36-fold, whereas Pro and other cluster 5 and 7 metabolites increased in HP and BH. This result indicates that metabolic regulation of osmotic stress occurring with CP and WD are fundamentally different from those induced by HP and BH. It is possible that sampling of CP and WD, which occurred at 5 and 2 h respectively after each stress was initiated, may be capturing very early phase metabolic perturbations before responsive gene transcription can have an effect. This would be especially likely if CP and WD elicit relatively slow responses. HP and BH are known to elicit relatively faster responses and were sampled at later times after the stress is applied (8.5 and 5 h respectively). It is likely that in the earliest phase of a stress response, as was sampled under CP and WD conditions, production of osmotic regulating metabolites by anabolic processes cannot be increased as rapidly as can catabolic production of maltose from starch, which provides a fundamentally different osmotic regulatory strategy (Thalmann and Santelia**,** 2017). This phenomenon also accounts for the single largest difference between BC and CP conditions with the pretreatment presumably triggering the production of β-amylase with maltose returning to more typical levels after recovery. In contrast, the BC stressed plants show much less dramatic changes in overall metabolite abundance after the initial stress is applied but fail to recover as quickly as the pretreated plants.

### Superimposing metabolite stress responses on pathways in central metabolism

**Fig. 4:**
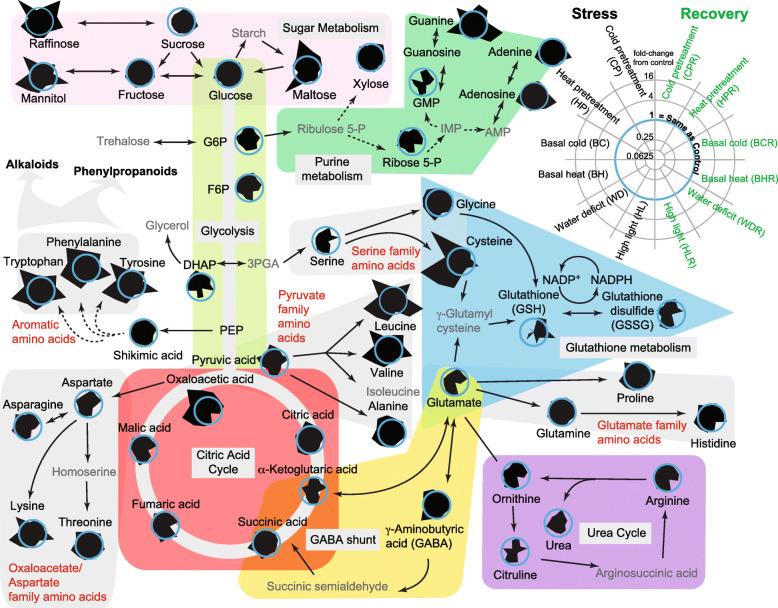
Metabolic pathways with radial plots showing the treatment effects on each metabolite observed. Radial plots were generated using the ratio change in abundance between treatment and control conditions for each metabolite. The blue circle indicates a ratio change of 1 where the control and treatment are identical. Treatment position in the radial plots is given in the key

We monitored metabolic pathways from several precursors and intermediates to products simultaneously to help reveal the subtle interplay of functionally related metabolites. Substrate and product relationships of identified metabolites that play critical roles in metabolism, stress tolerance, priming, and recovery processes are shown in Fig. 4. The radial plots for the individual metabolites can be easily visually compared to quickly scan metabolism for common or unique stress response patterns (larger versions of the radial plots used in Fig. 4 are provided in Supplemental Fig. S3). For example, the unique patterns of Maltose, Cys, and GSH are easy to visually identify as distinct from other metabolites. The bilateral symmetry of the plots also makes asymmetries as differences between stress and recovery. Sucrose, for example, shows a modest reduction in abundance for all the stress conditions but is similar to or above the control for the recovery conditions. Conversely, the mushroom-like symmetry of DHAP radial plot makes it easy to see that WD and WDR cause large decreases in the abundance of this metabolite and are close to the control for the other stresses. Glucose, fructose, glutamine and shikimic acid show remarkably subtle changes in abundance across all conditions that demonstrate the maintenance of homeostasis for some metabolic segments. Figure 4 also shows the important transitions that affect the stress and recovery processes as differences in radial plots help identify points in pathways where large perturbations occur such as in the TCA cycle where oxaloacetic acid increases dramatically under CP, HP, BC, BH and WD unlike any of the other pathway intermediates. There are many varied possible explanations for changes in metabolite abundance, from changes in levels of catalytic proteins, feedback inhibition, and rates of catabolism. It is remarkable to see the similarity in radial plots of amino acids Tyr, Phe, Trp, Leu, Val, and Lys despite coming from different segments of metabolism. This observation supports the notion of autolytic processes such as protein degradation, starch degradation (to maltose) as being important in early stress responses for producing ROS and osmoprotective metabolites prior to the induction of stress response genes (Hildebrandt, 2018). It is presumably easier or faster to activate the function of autolytic enzymes during initial periods of physical stress than to orchestrate the synthesis of protective metabolites through anabolic processes.

## Materials and methods

### Plant materials, growth conditions, and treatments

*Arabidopsis thaliana* wild-type (Col-0) seeds were obtained from Lehle Seeds and were sterilized with 30% (v/v) bleach containing 0.1% (v/v) Triton X-100 and vernalized at 4 °C for 2 days. Seedlings were germinated on 1/2 x Murashige and Skoog media made from Murashige and Skoog salts (PhytoTechnology Laboratories, M524 Murashige and Skoog Basal Salt Mixture, 4.33 g/L for 1 x MS) with 1.5% agar. Plants were grown prior to stress, during recovery, and as unstressed controls, vertically, at 22 °C under a 16-h-light/8-h-dark photoperiod, with ∼80 μmol m^− 2^ s^− 1^ illumination from cool-white fluorescent tube lights (control conditions). Eleven-day-old seedlings were treated with abiotic stress conditions described in Table 1. After the stress treatments, plants were removed back to the ‘control conditions’ for 2 days. Whole seedlings were harvested for the stress and recovery groups, frozen in liquid nitrogen, and stored at − 80 °C. Experiments were performed using three biological replicates, and each replicate consisted of 25 seedlings grown together on a single plate and pooled. The recovery group for water-deficit stress was obtained by moving the desiccated seedlings back to the agar medium under ‘control conditions’ for 2 days prior to harvest. Seedling hypocotyl length and root length were measured using *ImageJ* software (Schneider et al. 2012). ANOVA was used for calculating statistical differences.

### Sample extraction and LC-HRMS analysis

About 100 mg (fresh weight) of frozen seedlings were ground in 1 mL of 70% isopropanol alcohol with one 2.5 mm tungsten carbide ball in a Geno/Grinder™ (OPS Diagnostics) for 7 min at an intensity of 10,956 *x g* on − 20 °C chilled blocks. After centrifugation for 5 min at 100,956 *x g*, 1 μL of supernatant was injected onto a UHPLC (Ultimate 3000, Dionex) ZIC-cHILIC column (100 mm × 2.1 mm, 3 μm particle size, EMD Millipore Corporation, Billerica, MA). Solvents A (0.1% (v/v) formic acid in water) and B (0.1% (v/v) formic acid in acetonitrile) were used as mobile phases for gradient separation. Extract corresponding to 100 μg of plant material (fresh weight) was loaded onto the column with at a flow rate of 0.4 mL/min, followed by the following gradient separation: 2 min from 98% B, 30 min to 55% B, 2 min to 95% B and maintained for 3 min. The column was equilibrated for 2 min with 98% B prior to the next run. The samples were analyzed using a hybrid quadrupole Orbitrap mass spectrometer (Q Exactive, Thermo Fisher Scientific, San Jose CA). Full scan MS (range 50–750 *m/z*) were acquired with 35 k resolution for both positive and negative mode in polarity switching. The target value based on predictive automatic gain control was 1.0 × 10^6^ with 200 ms of maximum injection time. The flow rate for sheath gas, aux gas, and sweep gas was 50, 20, and 1 separately. The capillary temperature was 350 °C. The S-lens RF was set to 55. The Aux gas heater temperature was 300 °C. Targeted MS/MS acquisition was used during metabolite identification. The precursor ions were sequentially fragmented in the HCD collision cell with normalized collision energy of 10%, 20%, 40%. MS/MS scans were acquired with 17.5 k resolution and the target value was 2.0 × 10^5^ with 100 ms of maximum injection time. An isolation width of 2.0 *m/z* was used for precursor ion selection in MS/MS mode. GSH and GSSG were quantified by LC-HRMS using an external standard curve with 5, 50, 500 nM of each standard. The LC-HRMS analysis was carried out using the same method described above. The final concentration of GSH and GSSG in the injected solutions was calculated using peak areas and response factors calculated from the standard curves.

### Data analysis

Thermo RAW files were converted to *mzXML* format using *MSConvert* (Chambers et al., 2012). RAW files and *mzXML* files are available at MetaboLights (https://www.ebi.ac.uk/metabolights/) as MTBLS3672. Peak detection, grouping, and retention time correction were performed in *XCMS* in *R*. Statistics analysis including multivariate modeling PCA, OPLS-DA and hierarchical clustering were performed in Workflow4Metabolomics.org online resource for computational metabolomics (Giacomoni et al., 2015). Annotation was first performed by databases including *BMRB* (Markley et al., 2008), *METLIN* (Smith et al., 2005), and *HMDB* (Wishart et al., 2013) public databases, and identities were confirmed with authentic standards for *m/z*, retention time, and fragmentation data listed in Supplemental Table S2. Univariate testing by ANOVA was performed using both the Workflow4Metabolomics.org online resource (Giacomoni et al., 2015) and Excel 2016 (Microsoft). Fold-changes in intensities for metabolites observed in both ionization modes were averaged if they were deemed indistinguishable by ANOVA, otherwise the values derived from the most intense signals were used on the assumption that the values derived from lower signals were so due to ion-suppression. The peak area for each targeted metabolite was exported from the *Quan Browser* module of the *Xcalibur* software (Thermo Fisher Scientific) after visual confirmation of mass and retention times to validate the *XCMS* feature extraction. Metabolic pathways were generated by reference to the *KEGG* pathway Database (Kanehisa et al., 2017). Radial plots were generated using *KaleidaGraph*. Metabolites as potential stress metabolic signatures and their corresponding precursors, intermediates, or products in the same metabolic pathway that were identified by authentic standards with *m/z*, retention time, and fragmentation (Supplemental Fig. S2.1, Supplemental Fig. S2.2, Supplemental Table S2).

## Supplementary Information

**Additional file 1: Supplemental Figure S1.** (A) Average fresh weight, (B) hypocotyl length, (C) root length of 11-day-old seedlings (*n* = 25 seedlings per treatment) grown on medium with stress treatment before and after 2-day-recovery. Error bars represent standard deviation. Asterisks indicate significant differences (*: *P* < 0.05; **: *P* < 0.01) by ANOVA. **Supplemental Figure S2.1.** Metabolites that were identified by authentic standards with *m/z*, retention time, and fragmentation in positive mode. The top scheme represents metabolite in sample. The bottom scheme represents metabolite in standard. Standard compounds include: adenine (A), adenosine (B), γ-aminobutyric acid (C), GSH (D), GSSG (E), guanine (F), guanosine monophosphate (G), L-alanine (H), L-arginine (I), L-asparagine (J), L-aspartic acid (K), L-citrulline (L), L-cysteine (M), L-glutamic acid (N), L-glutamine (O), L-glycine (P), L-histidine (Q), L-leucine (R), L-lysine (S), L-ornithine (T), L-phenylalanine (U), L-proline (V), L-serine (W), L-threonine (X), L-tryptophan (Y), L-tyrosine (Z), L-valine (AA), mannitol (AB), urea (AC). It should be noted that while standard compounds of the specified stereochemistry were used, analytical procedures that would resolve enantiomers were not employed. Because of this absolute stereo-assignments for each of these compounds were not included as part of the assignment, though it is likely that the vast majority of amino acids identified are of the L-configuration as D-amino acids are unusual in plants and typically occur in specialized metabolic contexts. **Figure S2.2.** Metabolites that were identified by authentic standards with *m/z*, retention time, and fragmentation in negative mode. The top scheme represents metabolite in sample. The bottom scheme represents metabolite in standard. Standard compounds include: adenosine (A), α-ketoglutaric acid (B), citric acid (C), dihydroxyacetone phosphate (D), fructose (E), fructose 6-phosphate (F), fumaric acid (G), glucose (H), glucose 6-phosphate (I), GSH (J), GSSG (K), L-arginine (L), L-asparagine (M), L-aspartic acid (N), L-citrulline (O), L-glutamic acid (P), L-glutamine (Q), L-glycine (R), L-histidine (S), L-leucine (T), L-lysine (U), L-ornithine (V), L-phenylalanine (W), L-serine (X), L-threonine (Y), L-tryptophan (Z), L-tyrosine (AA), L-valine (AB), malic acid (AC), maltose (AD), mannitol (AE), oxaloacetate (AF), pyruvic acid (AG), raffinose (AH), ribose 5-phosphate (AI), shikimic acid (AJ), succinic acid (AK), sucrose (AL), xylose (AM). As noted above, while standard compounds of the specified stereochemistry were used, analytical procedures that would resolve enantiomers were not employed.

**Additional file 2: Supplemental Figure S3.** Detailed radial plots for each individual metabolite shown in Fig. 4. The plots are presented in the seven clusters and order derived from hierarchical clustering as presented in Fig. 2.

**Additional file 3: Supplemental Table S1.** Summary of previous metabolomics studies of plant responses to stress caused by heat, cold, freezing, water-deficit, and high light exposure. The table provides a reference to each report, the plant species, tissue studied, and the analytical approach used for each study.

**Additional file 4: Supplemental Table S2.** Metabolites identified by comparison of chromatographic retention, accurate mass, and MS/MS fragmentation patterns with those of reference standards. **Supplemental Table S3.** Average fold change for metabolites compared to control under stress and recovery treatments. **Supplemental Table S4.** Standard compounds used for feature identification by retention time and MS/MS fragmentation pattern matching. **Supplemental Table S5.** Unidentified metabolic features significantly increased or decreased in responses to different treatments (greater than 2-fold change from control, significance tested using ANOVA with *P* < 0.05).

## Data Availability

Data used for metabolite identification and untargeted metabolic feature data are provided in the Supplementary files. Raw data files generated during and/or analyzed during the current study (.RAW and mzML formats) as well as the corresponding metabolomics data matrix have been deposited in the MetaboLights metabolomics database (https://www.ebi.ac.uk/metabolights/) under accession# MTBLS3672.

## Abbreviations

BC: Basal cold stress
BCR: Basal cold recovery
BH: Basal heat stress
BHR: Basal heat recovery
CP: Cold pretreatment stress
CPR: Cold pretreatment recovery
DHAP: Dihydroxyacetone phosphate
GC-MS: Gas chromatography–mass spectrometry
GMP: Guanosine monophosphate
GSH: Glutathione
GSSG: Glutathione disulfide
GSTs: Glutathione S-transferases
HCA: Hierarchical clustering analysis
HCD: Higher-energy C-trap dissociation
HILIC: Hydrophilic interaction liquid chromatography
HL: High light stress
HLR: High light recovery
HP: Heat pretreatment stress
HPLC: High performance liquid chromatography
HPR: Heat pretreatment recovery
LC-HRMS: Liquid chromatography–high resolution mass spectrometry
MS: Mass spectrometry
MS/MS: Tandem mass spectrometry
OPLS: orthogonal projection to latent structures
OPLS-DA: Orthogonal projections to latent structures discriminant analysis
OSC: Orthogonal signal correction
PCA: Principal component analysis
ROS: Reactive oxygen species
TCA: Tricarboxylic acid
UHPLC: Ultra high performance liquid chromatograph
WD: Water-deficit stress
WDR: Water-deficit recovery
