# Supplementary material for: Metabolic signatures of Arabidopsis thaliana abiotic stress responses elucidate patterns in stress priming, acclimation, and recovery
Source: Stress Biol. 2022 Feb 15;2(1):11. doi: 10.1007/s44154-022-00034-5 (PMC10441859; doi:10.1007/s44154-022-00034-5)
Supplement: Supplementary file 1 — Additional file 1: Supplemental Figure S1. (A) Average fresh weight, (B) hypocotyl length, (C) root length of 11-day-old seedlings (n = 25 seedlings per treatment) grown on medium with stress treatment before and after 2-day-recovery. Error bars represent standard deviation. Asterisks indicate significant differences (*: P < 0.05; **: P < 0.01) by ANOVA. Supplemental Figure S2.1. Metabolites that were identified by authentic standards with m/z, retention time, and fragmentation in positive mode. The top scheme represents metabolite in sample. The bottom scheme represents metabolite in standard. Standard compounds include: adenine (A), adenosine (B), γ-aminobutyric acid (C), GSH (D), GSSG (E), guanine (F), guanosine monophosphate (G), L-alanine (H), L-arginine (I), L-asparagine (J), L-aspartic acid (K), L-citrulline (L), L-cysteine (M), L-glutamic acid (N), L-glutamine (O), L-glycine (P), L-histidine (Q), L-leucine (R), L-lysine (S), L-ornithine (T), L-phenylalanine (U), L-proline (V), L-serine (W), L-threonine (X), L-tryptophan (Y), L-tyrosine (Z), L-valine (AA), mannitol (AB), urea (AC). It should be noted that while standard compounds of the specified stereochemistry were used, analytical procedures that would resolve enantiomers were not employed. Because of this absolute stereo-assignments for each of these compounds were not included as part of the assignment, though it is likely that the vast majority of amino acids identified are of the L-configuration as D-amino acids are unusual in plants and typically occur in specialized metabolic contexts. Figure S2.2. Metabolites that were identified by authentic standards with m/z, retention time, and fragmentation in negative mode. The top scheme represents metabolite in sample. The bottom scheme represents metabolite in standard. Standard compounds include: adenosine (A), α-ketoglutaric acid (B), citric acid (C), dihydroxyacetone phosphate (D), fructose (E), fructose 6-phosphate (F), fumaric acid (G), glucose (H), gl [file 44154_2022_34_MOESM1_ESM.pdf]

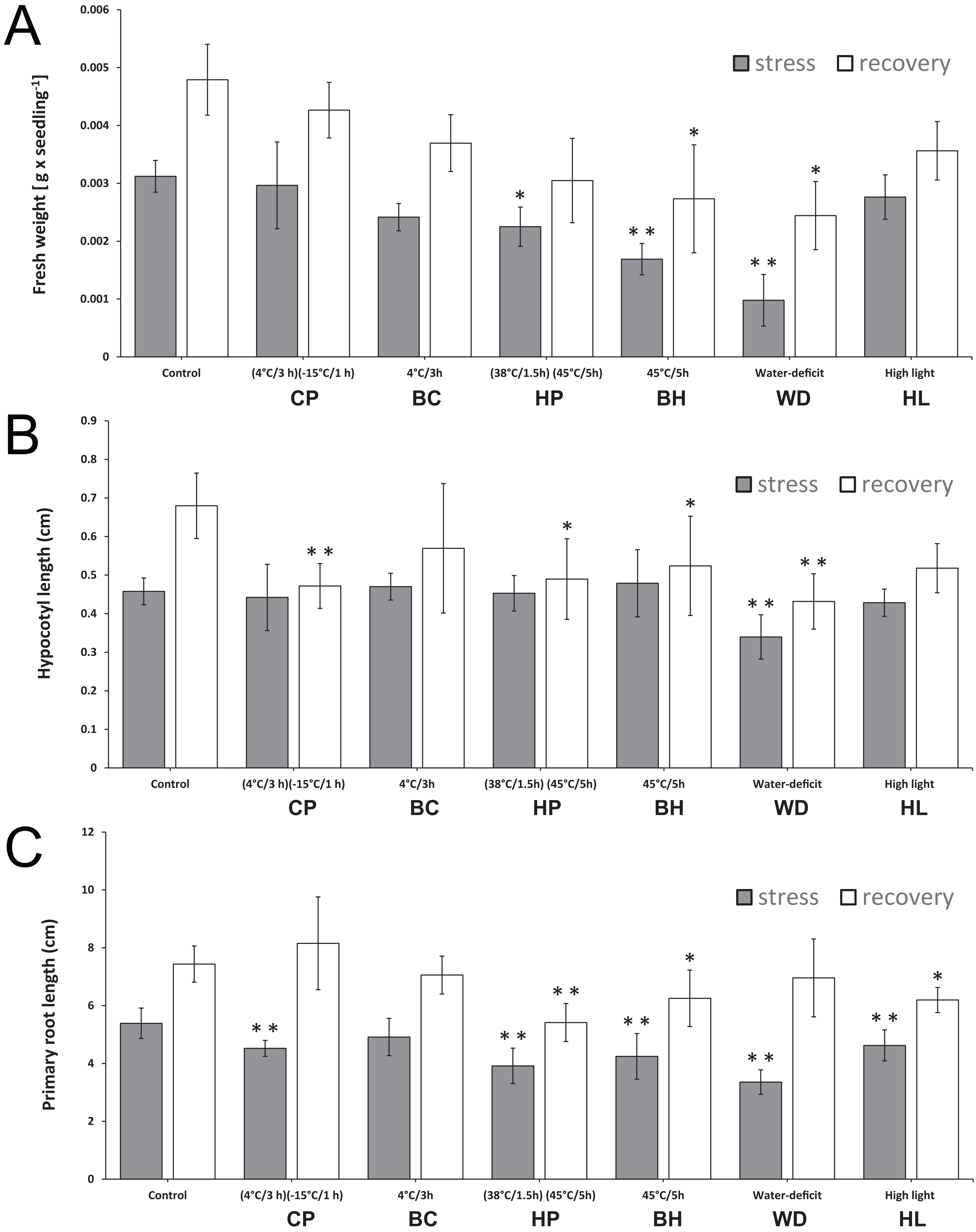

Supplemental Figure S1. (A) Average fresh weight, **(B)** hypocotyl length, **(C)** root length of 11-day-old seedlings (n = 25 seedlings per treatment) grown on medium with stress treatment before and after 2-day-recovery. Error bars represent standard deviation. Asterisks indicate significant differences (*: $P < 0.05$; **: $P < 0.01$) by ANOVA.

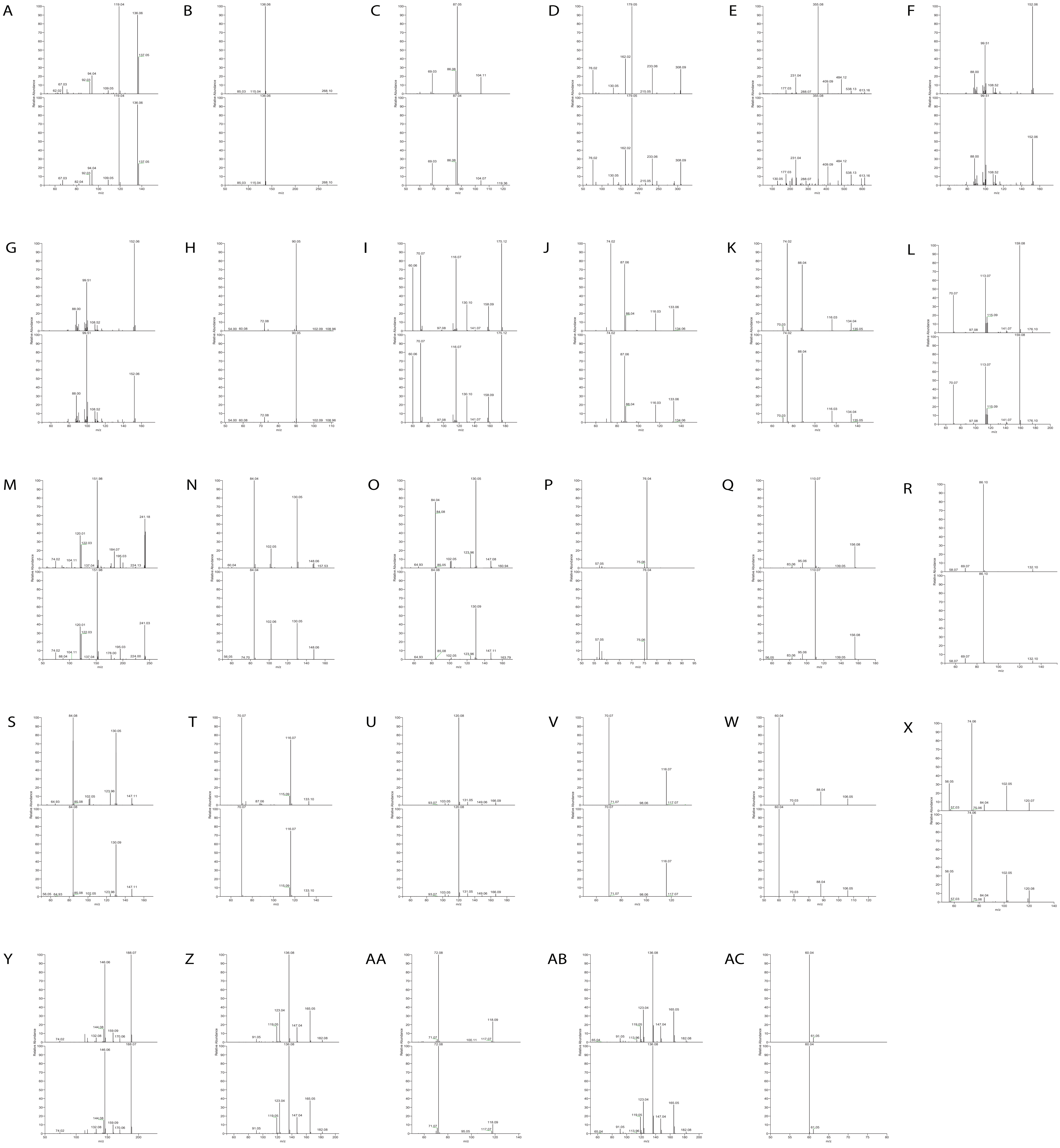

Supplemental Figure S2.1. Metabolites that were identified by authentic standards with m/z, retention time, and fragmentation in positive mode. The top scheme represents metabolite in sample. The bottom scheme represents metabolite in standard. Standard compounds include: adenine (A), adenosine (B), γ-aminobutyric acid (C), GSH (D), GSSG (E), guanine (F), guanosine monophosphate (G), L-alanine (H), L-arginine (I), L-asparagine (J), L-aspartic acid (K), L-citrulline (L), L-cysteine (M), L-glutamic acid (N), L-glutamine (O), L-glycine (P), L-histidine (Q), L-leucine (R), L-lysine (S), L-ornithine (T), L-phenylalanine (U), L-proline (V), L-serine (W), L-threonine (X), L-tryptophan (Y), L-tyrosine (Z), L-valine (AA), mannitol (AB), urea (AC). It should be noted that while standard compounds of the specified stereochemistry were used, analytical procedures that would resolve enantiomers were not employed. Because of this absolute stereo-assignments for each of these compounds were not included as part of the assignment, though it is likely that the vast majority of amino acids identified are of the L-configuration as D-amino acids are unusual in plants and typically occur in specialized metabolic contexts.

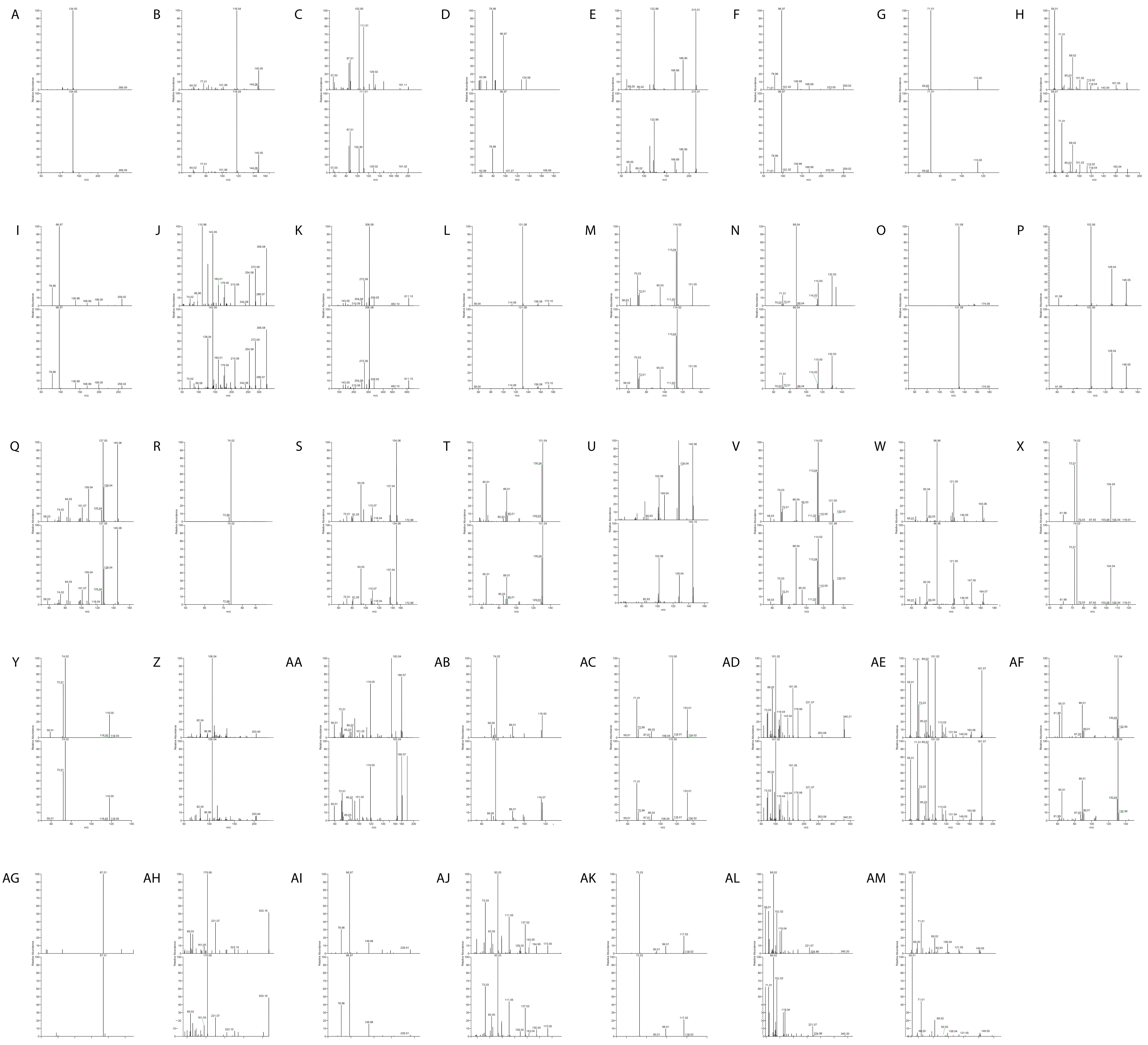

Supplemental Figure S2.2. Metabolites that were identified by authentic standards with m/z, re-tention time, and fragmentation in negative mode. The top scheme represents metabolite in sample. The bottom scheme represents metabolite in standard. Standard compounds include: adenosine (A), α-ketoglutaric acid (B), citric acid (C), dihydroxyacetone phosphate (D), fructose (E), fructose 6-phosphate (F), fumaric acid (G), glucose (H), glucose 6-phosphate (I), GSH (J), GSSG (K), L-arginine (L), L-asparagine (M), L-aspartic acid (N), L-citrulline (O), L-glutamic acid (P), L-glutamine (Q), L-glycine (R), L-histidine (S), L-leucine (T), L-lysine (U), L-ornithine (V), L-phenylalanine (W), L-serine (X), L-threonine (Y), L-tryptophan (Z), L-tyrosine (AA), L-valine (AB), malic acid (AC), maltose (AD), mannitol (AE), oxaloacetate (AF), pyruvic acid (AG), raffinose (AH), ribose 5-phosphate (AI), shikimic acid (AJ), succinic acid (AK), sucrose (AL), xylose (AM). As noted above, while standard compounds of the specified stereochemistry were used, analytical procedures that would resolve enantiomers were not employed.
