# Supplementary material for: Metabolic signatures of Arabidopsis thaliana abiotic stress responses elucidate patterns in stress priming, acclimation, and recovery
Source: Stress Biol. 2022 Feb 15;2(1):11. doi: 10.1007/s44154-022-00034-5 (PMC10441859; doi:10.1007/s44154-022-00034-5)
Supplement: Supplementary file 3 — Additional file 3: Supplemental Table S1. Summary of previous metabolomics studies of plant responses to stress caused by heat, cold, freezing, water-deficit, and high light exposure. The table provides a reference to each report, the plant species, tissue studied, and the analytical approach used for each study. [file 44154_2022_34_MOESM3_ESM.pdf]

[1]**Supplemental Table S1.**

Stress	References	Plant species	Tissue of interest	Analytical approach
heat	Kaplan et al., 2004	*Arabidopsis thaliana*	aerial tissues	GC-MS
	Luengwilai et al., 2012	*Solanum lycopersicum*	fruit pericarp	GC-MS
	Sun et al., 2016	*Zea mays*	leaf	NMR
	Paupière et al., 2017	*S. lycopersicum*	pollen	LC–QTOF–MS
	Das et al., 2017	*Glycine max*	leaf	UPLC-MS/MS
	Sang et al., 2017	*Lycopersicon esculentum*	seedling	MALDI-TOF/TOF
	Thomason et al., 2018	*Triticum aestivum*	leaf	LC/HRMS
	Wang et al., 2018	*T. aestivum*	grain	LC-MS/MS
	Paupière et al., 2020	*S. lycopersicum*	leaf	LC-QTOF-MS
cold	Kaplan et al., 2007	*A. thaliana*	aerial tissues	GC-MS
	Welti et al., 2002	*A. thaliana*	rosettes	LC-MS/MS
	Kaplan et al., 2004	*A. thaliana*	aerial tissues	GC-MS
	Gray and Heath, 2005	*A. thaliana*	seedlings	LC-MS
	Caldana et al., 2011	*A. thaliana*	rosettes	GC-MS
	Guevara et al., 2012	*Thellungiella salsuginea*	leaf	GC-MS
	Benina et al., 2013	*Haberlea rhodopensis, Thellungiella halophyla,* and *A. thaliana*	leaf	GC-MS
	Zhao et al., 2019	*T. aestivum*	seedlings	LC-MS
freezing	Hannah, 2006	*A. thaliana*	leaf	GC/TOFMS
	Korn et al., 2010	*A. thaliana*	leaf	GC-MS
water-deficit	Urano et al., 2009	*A. thaliana*	aerial tissues	GC/TOFMS, CE-MS
	Yang et al., 2007	*Oryza sativa*	leaf	HPLC
	Antonio et al., 2008	*Lupinus albus*	stem	PGC-LCESI-MS
	Skirycz et al., 2010	*A. thaliana*	leaf	GC/TOFMS

	Bowne et al., 2012	*T. aestivum*	leaf	GC-MS
	Erxleben et al., 2012	*Physcomitrium patens*	Multiple tissues	GC-MS
	Witt et al., 2012	*Z. mays*	Multiple tissues	GC-TOFMS
	Sicher and Barnaby, 2012	*Z. mays*	leaf	UPLC-GC/MS
	Gechev et al., 2013	*H. rhodopensis*	leaf	GC-MS, LC-MS
	Wang et al., 2021	*Poa crymophila* Keng	seeds	LC-MS
high light exposure	Wulff-Zottele et al., 2010	*A. thaliana*	leaf	GC-MS
	Caldana et al., 2011	*A. thaliana*	rosettes	GC-MS
	Küstner et al., 2019	*A. thaliana*	rosettes	LC-MS/MS

[1]Supplemental Table S1 summarizes previous metabolomics studies of plant responses to stress caused by heat, cold, freezing, water-deficit, and high light exposure. The table provides a reference to each report, the plant species, tissue studied, and the analytical approach used for the analysis.

References:

Antonio, C., Pinheiro, C., Chaves, M.M., Ricardo, C.P., Ortuño, M.F. and Thomas-Oates, J. (2008) Analysis of carbohydrates in Lupinus albus stems on imposition of water deficit, using porous graphitic carbon liquid chromatography-electrospray ionization mass spectrometry. *J. Chromatogr. A*. **1187(1-2)**, 111-118. Available at: https://doi.org/10.1016/j.chroma.2008.02.010

Benina, M., Obata, T., Mehterov, N., Ivanov, I., Petrov, V., Toneva, V., Fernie, A.R. and Gechev, T.S. (2013) Comparative metabolic profiling of *Haberlea rhodopensis*, *Thellungiella halophyla*, and *Arabidopsis thaliana* exposed to low temperature. *Front. Plant Sci.* **4**, 499 Available at: https://doi.org/10.3389/fpls.2013.00499

Bowne, J.B., Erwin, T.A., Juttner, J., Schnurbusch, T., Langridge, P., Bacic, A. and Roessner, U. (2012) Drought responses of leaf tissues from wheat cultivars of differing drought tolerance at the metabolite level. *Molecular Plant* **5(2)**:418-29. Available at: https://doi.org/10.1093/mp/ssr114

Caldana, C., Degenkolbe, T., Cuadros-Inostroza, A., Klie, S., Sulpice, R., Leisse, A., Steinhauser, D., Fernie, A.R., Willmitzer, L. and Hannah, M.A. (2011) High-density kinetic analysis of the metabolomic and transcriptomic response of Arabidopsis to eight environmental conditions. *Plant J.* **67**, 869–884. Available at: https://doi.org/10.1111/j.1365-313X.2011.04640.x

Das, A., Rushton, P.J. and Rohila, J.S. (2017) Metabolomic profiling of soybeans (*Glycine max* L.) reveals the importance of sugar and nitrogen metabolism under drought and heat stress. *Plants (Basel, Switzerland)* **6(2)**, 21. Available at: https://doi.org/10.3390/plants6020021

Erxleben, A., Gessler, A., Vervliet-Scheebaum, M. and Reski, R. (2012) Metabolite profiling of the moss *Physcomitrella patens* reveals evolutionary conservation of osmoprotective substances. *Plant Cell Rep.* **31(2)**, 427-436 Available at: https://doi.org/10.1007/s00299-011-1177-9

Gechev, T.S., Benina, M., Obata, T., Tohge, T., Sujeeth, N., Minkov, I., Hille, J., Temanni, M.R., Marriott, A.S., Bergström, E., Thomas-Oates, J., Antonio, C., Mueller-Roeber, B., Schippers, J.H., Fernie, A.R., and Toneva, V. (2013) Molecular mechanisms of desiccation tolerance in the resurrection glacial relic *Haberlea rhodopensis*. *Cell Mol Life Sci.* **70(4)**, 689-709. Available at: https://doi.org/10.1007/s00018-012-1155-6

Gray, G.R. and Heath, D. (2005) A global reorganization of the metabolome in Arabidopsis during cold acclimation is revealed by metabolic fingerprinting. *Physiol. Plant.* **124**, 236–248. Available at: https://doi.org/10.1111/j.1399-3054.2005.00507.x

Guevara, D. R., Champigny, M. J., Tattersall, A., Dedrick, J., Wong, C. E., Li, Y., Labbe, A., Ping, C. L., Wang, Y., Nuin, P., Golding, G. B., McCarry, B. E., Summers, P. S., Moffatt, B. A., and Weretilnyk, E. A. (2012) Transcriptomic and metabolomic analysis of Yukon *Thellungiella* plants grown in cabinets and their natural habitat show phenotypic plasticity. *BMC Plant Biol.* **12**, 175. Available at: *https://doi.org/10.1186/1471-2229-12-175*

Hannah, M.A. (2006) Natural genetic variation of freezing tolerance in Arabidopsis. *Plant Physiol.* **142**, 98–112. Available at: https://doi.org/10.1104/pp.106.081141.

Kaplan, F., Kopka, J., Haskell, D.W., Zhao, W., Schiller, K.C., Gatzke, N., Sung, D.Y. and Guy, C.L. (2004) Exploring the temperature-stress metabolome. *Plant Physiol.* **136**, 4159–4168. Available at: https://doi.org/10.1104/pp.104.052142

Kaplan, F., Kopka, J., Sung, D.Y., Zhao, W., Popp, M., Porat, R. and Guy, C.L. (2007) Transcript and metabolite profiling during cold acclimation of Arabidopsis reveals an intricate relationship of cold-regulated gene expression with modifications in metabolite content. *Plant J.* **50**, 967–981. Available at: https://doi.org/10.1111/j.1365-313X.2007.03100.x

Korn, M., Gärtner, T., Erban, A., Kopka, J., Selbig, J. and Hincha, D.K. (2010) Predicting Arabidopsis freezing tolerance and heterosis in freezing tolerance from metabolite composition. *Mol. Plant* **3**, 224–235. Available at: http://dx.doi.org/10.1093/mp/ssp105.

Küstner, L., Fürtauer, L., Weckwerth, W., Nägele, T. and Heyer, A.G. (2019) Subcellular dynamics of proteins and metabolites under abiotic stress reveal deferred response of the *Arabidopsis thaliana* hexokinase-1 mutant gin2-1 to high light. *Plant J.* **100(3)**, 456-472. Available at: https://doi.org/10.1111/tpj.14491

Luengwilai, K., Saltveit, M. and Beckles, D.M. (2012) Metabolite content of harvested Micro-Tom tomato (*Solanum lycopersicum* L.) fruit is altered by chilling and protective heat-shock treatments as shown by GC-MS metabolic profiling. *Postharvest Biol. Technol.* **63(1)**, 116-122 Available at: https://doi.org/10.1016/j.postharvbio.2011.05.014

Paupière, M.J., Müller, F., Li, H., Rieu, I., Tikunov, Y.M., Visser, R.G.F. and Bovy, A.G. (2017) Untargeted metabolomic analysis of tomato pollen development and heat stress response. *Plant Reprod.* **30(2)**, 81-94. Available at: https://doi.org/10.1007/s00497-017-0301-6

Paupière, M.J., Tikunov, Y., Schleiff, E., Bovy, A. and Fragkostefanakis, S. (2020) Reprogramming of tomato leaf metabolome by the activity of heat stress transcription factor HsfB1. *Front. Plant Sci.* **11**, 610599 Available at: https://doi.org/10.3389/fpls.2020.610599

Sang, Q., Shan, X., An, Y., Shu, S., Sun, J. and Guo, S. (2017) Proteomic analysis reveals the positive effect of exogenous spermidine in tomato seedlings' response to high-temperature stress. *Front. Plant Sci.* **8**, 120. Available at: https://doi.org/10.3389/fpls.2017.00120

Sicher, R.C. and Barnaby, J.Y. (2012) Impact of carbon dioxide enrichment on the responses of maize leaf transcripts and metabolites to water stress. *Physiol. Plant.* **144(3)**, 238-53. Available at: https://doi.org/10.1111/j.1399-3054.2011.01555.x.

Skirycz, A., De Bodt, S., Obata, T., De Clercq, I., Claeys, H., De Rycke, R., Andriankaja, M., Van Aken, O., Van Breusegem, F., Fernie, A.R. and Inzé, D. (2010) Developmental stage specificity and the role of mitochondrial metabolism in the response of Arabidopsis leaves to prolonged mild osmotic stress. *Plant Physiol.* **152**, 226–244. Available at: https://doi.org/10.1104/pp.109.148965.

Sun, C.X., Gao, X.X., Li, M.Q., Fu, J.Q. and Zhang, Y.L. (2016) Plastic responses in the metabolome and functional traits of maize plants to temperature variations. *Plant Biol.* **18(2)**, 249-261 Available at: https://doi.org/10.1111/plb.12378

Thomason, K., Babar, M.A., Erickson, J.E., Mulvaney, M., Beecher, C. and MacDonald, G. (2018) Comparative physiological and metabolomics analysis of wheat (*Triticum aestivum* L.) following post-anthesis heat stress. *PLoS One* **13(6)**, e0197919. Available at: https://doi.org/10.1371/journal.pone.0197919

Urano, K., Maruyama, K., Ogata, Y., Morishita, Y., Takeda, M., Sakurai, N., Suzuki, H., Saito, K., Shibata, D., Kobayashi, M., Yamaguchi-Shinozaki, K. and Shinozaki, K. (2009) Characterization of the ABA-regulated global responses to dehydration in Arabidopsis by metabolomics. *Plant J.* **57**, 1065–1078. Available at: https://doi.org/10.1111/j.1365-313X.2008.03748.x

Wang, X., Hou, L., Lu, Y., Wu, B., Gong, X., Liu, M., Wang, J., Sun, Q., Vierling, E. and Xu, S. (2018) Metabolic adaptation of wheat grain contributes to a stable filling rate under heat stress. *J. Exp. Bot.* **69(22)**, 5531-5545. Available at: https://doi.org/10.1093/jxb/ery303.

Wang, Y., Li, X.Y., Li, C.X., He, Y., Hou, X.Y. and Ma, X.R. (2021) The regulation of adaptation to cold and drought stresses in *Poa crymophila* Keng revealed by integrative transcriptomics and metabolomics analysis. *Front. Plant Sci.* **12,** 631117. Available at: https://doi.org/10.3389/fpls.2021.631117

Welti, R., Li, W., Li, M., Sang, Y., Biesiada, H., Zhou, H.-E., Rajashekar, C.B., Williams, T.D. and Wang, X. (2002) Profiling membrane lipids in plant stress responses. *J. Biol. Chem.* **277(35)**, 31994-2002. Available at: https://doi.org/10.1074/jbc.M205375200

Witt, S., Galicia, L., Lisec, J., Cairns, J., Tiessen, A., Araus, J.L., Palacios-Rojas, N. and Fernie, A.R. (2012) Metabolic and phenotypic responses of greenhouse-grown maize hybrids to experimentally controlled drought stress. *Mol. Plant* **5**, 401–417. Available at: http://dx.doi.org/10.1093/mp/ssr102.

Wulff-Zottele, C., Gatzke, N., Kopka, J., Orellana, A., Hoefgen, R., Fisahn, J. and Hesse, H. (2010) Photosynthesis and metabolism interact during acclimation of *Arabidopsis thaliana* to high irradiance and sulphur depletion. *Plant, Cell Environ.* **33**, 1974–1988. Available at: https://doi.org/10.1111/j.1365-3040.2010.02199.x

Yang, J., Zhang, J., Liu, K., Wang, Z. and Liu, L. (2007) Involvement of polyamines in the drought resistance of rice. *J. Exp. Bot.* **58(6)**, 1545-1555. Available at: https://doi.org/10.1093/jxb/erm032

Zhao, Y., Zhou, M., Xu, K., Li, J., Li, S., Zhang, S. and Yang, X. (2019) Integrated transcriptomics and metabolomics analyses provide insights into cold stress response in wheat. *Crop J.* **7(6)**, 857-866 Available at: https://doi.org/10.1016/j.cj.2019.09.002
