# Supplementary material for: Metabolic signatures of Arabidopsis thaliana abiotic stress responses elucidate patterns in stress priming, acclimation, and recovery
Source: Stress Biol. 2022 Feb 15;2(1):11. doi: 10.1007/s44154-022-00034-5 (PMC10441859; doi:10.1007/s44154-022-00034-5)

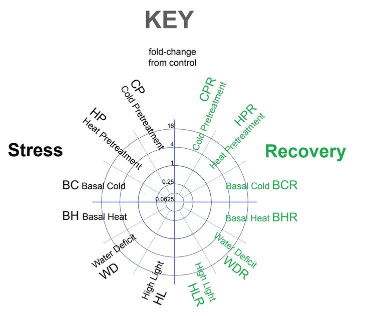

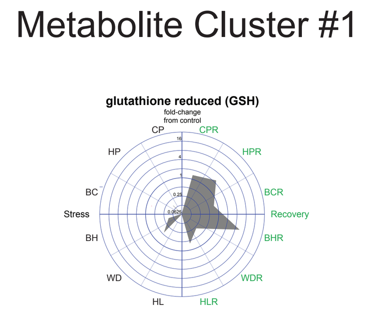

Supplemental Figure S3. Detailed radial plots for each individual metabolite shown in Figure 4. The plots are presented in the seven clusters and order derived from hierarchical clustering as presented in Figure 2.

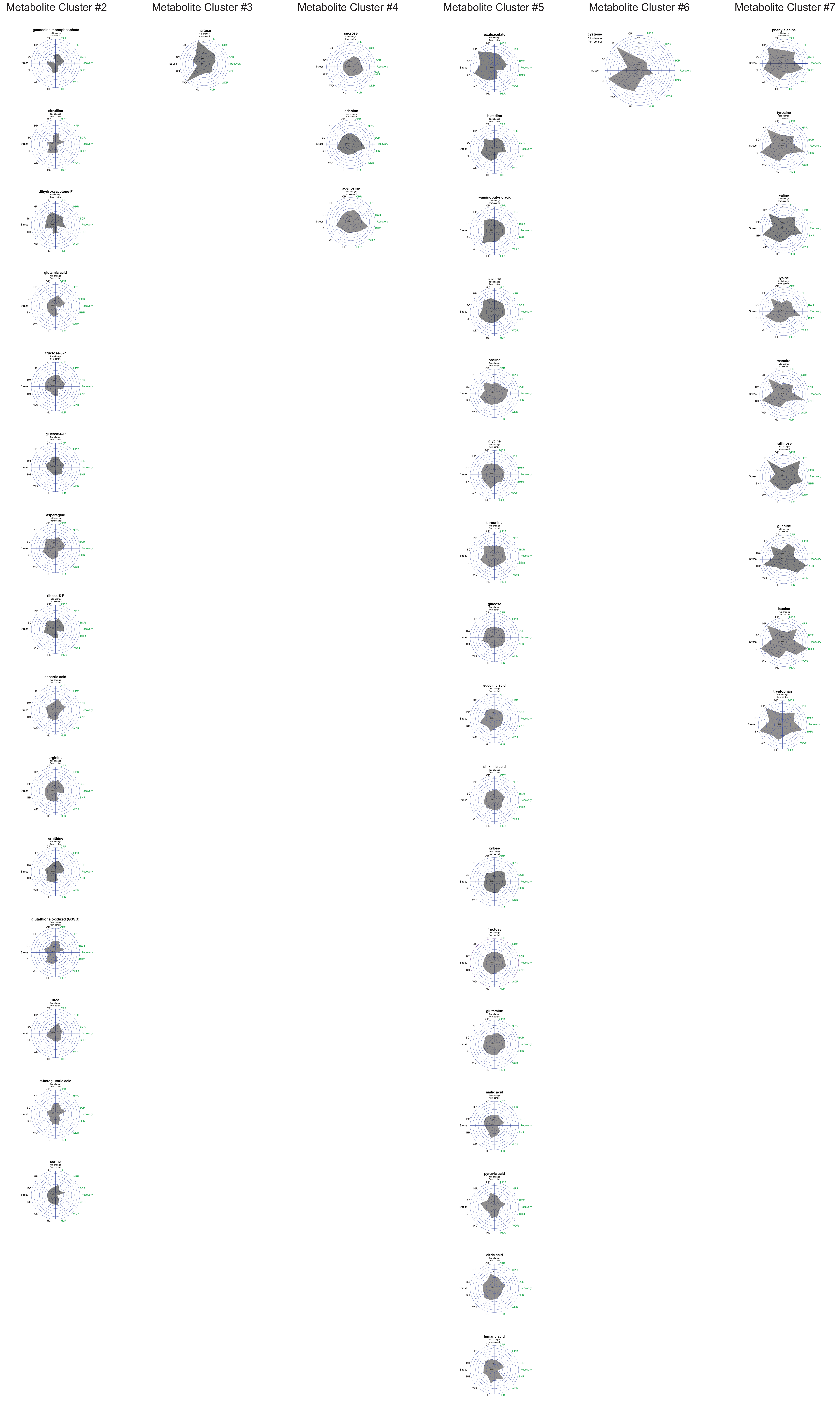

Supplement: Supplementary file 2 — Additional file 2: Supplemental Figure S3. Detailed radial plots for each individual metabolite shown in Fig. 4. The plots are presented in the seven clusters and order derived from hierarchical clustering as presented in Fig. 2. [file 44154_2022_34_MOESM2_ESM.pdf]
